# Effectiveness of a prevention programme of surgical site infection after major heart surgery

**DOI:** 10.1186/2197-425X-3-S1-A119

**Published:** 2015-10-01

**Authors:** M Nieto, M Sanchez, M Bringas, M Requesens, I García, A Del Pino, F Reguillo, C Fernandez

**Affiliations:** Intensive Care, Hospital Clinico San Carlos, Madrid, Spain; Hospital Clinico San Carlos, Madrid, Spain; Cardiac Surgerym, Hospital Clinico San Carlos, Madrid, Spain; Epidemiology Unit, Hospital Clinico San Carlos, Madrid, Spain

## Introduction

The surgical site infection (SSI) after heart surgery is infrecuent but is associated with significant morbidity and mortality with increased hospital stay particularly when mediastinitis occurs. Surveillance and infection control measures are important cornerstones to prevent SSI.

## Objectives

To evaluate the results after 42 months of implementation of multidisciplinary protocol for the prevention, diagnostic work-up and therapy of surgical site infection (SSI) in major heart surgery (MHS) in a tertiary university hospital.

## Methods

The checklist-guided bundle implements pre (topical decontamination, shaving, systemic prophylaxis), intra (hygiene, surgical technique, metabolic, temperature) and postoperative (wound care, physiotherapy, mechanical support of sternotomy, pain control) interventions. Outcome variables are rate of SSI, length of hospital stay and mortality. The outcome superficial SSI and “mediastinitis” was defined according to CDC criteria. The study cohort of patients in whom the protocol was applied over the first 42 months after implementation was compared to a historical control group of patients undergoing MHS over 78 months. Potential confounding factors of the effect of the intervention were considered calculating propensity score of pre, intra and postoperative variables. The impact of the protocol was estimated by relative risk reduction (RRR and CI95%) with univariate analysis, stratification and Poisson regression models. Mean costs of ICU and hospital stay were calculated. Statistical analyses were performed with STATA 12.0.

## Results

2304 patients (June 2011 to December 2014) were compared to 4317 patients (January 2005 to May 2011). Significant differences were observed in the incidence of obesity, duration of ischemia and extracorporeal circulation, previous mechanical ventilation and perioperative intra-aortic balloon pump. Adjusted relative risk of SSI was significantly reduced by 36% (RR 0.64; CI95% 0.42-0.97). Adjusted relative risk of overall mortality was significantly reduced by 41% (RR 0.59; CI95% 0.35-0.99). and hospital median length of stay was reduced around 50% (46 days (IQR 17-69) in front of 29 days (9-46) p < 0,001.

## Conclusions

The implementation of a multidisciplinary prevention protocol for SSI in MHS was associated with a significant reduction of mediastinitis risk, mortality and cost.
